# Protective Effect of Combined Long Time Administration of Selenium and Vitamin C on Liver and Kidney Toxicity of Cadmium in Rats

**DOI:** 10.30699/IJP.2020.135777.2489

**Published:** 2020-11-23

**Authors:** Mohammad Mahdi Zamani, Seyedeh Hamideh Mortazavi, Maryam Monajjemzadeh, Vahhab Piranfar, Zahra Aalidaeijavadi, Azam Bakhtiarian

**Affiliations:** 1 *Exceptional Talent Development Center (EDTC), Tehran University of Medical Sciences, Tehran, Iran*; 2 *Department of Anesthesiology and Critical Care, Hasheminejad Kidney Center (HKC), Iran University of Medical Sciences, Tehran, Iran *; 3 *Scientific Students’ Research Center, Tehran University of Medical Sciences, Tehran, Iran *; 4 *Department of Pathology, School of Medicine, Tehran University of Medical Sciences, Tehran, Iran *; 5 *Department of Medical Microbiology, School of Medicine, Iran University of Medical Science, Tehran, Iran*; 6 *Department of Pharmacology, School of Medicine, Tehran University of Medical Sciences, Tehran, Iran *

**Keywords:** Antioxidants, Ascorbic Acid, Selenium, Kidney, Liver

## Abstract

**Background & Objective:**

Increased industrial activities leads to prolonged human exposure to industrial pollutant such as cadmium (Cd). ‍Chronic exposure to Cd in Mammals and also human being, can cause damages to various organs and particularly kidneys and liver. The goal of this study was to investigate the prophylactic effects of combined selenium (Se) and ascorbic acid supplement in rat cadmium toxicity.

**Methods:**

Sixty adult male Wistar rats were divided to 10 groups: one control, one sham and two clusters of 4 intervention groups which were fed with 1 or 5 mg Cd /kg water, for 28 days. Ascorbic acid supplement was added to drinking water of four groups (10 mg/L). Four groups received intraperitoneal Se (1 mg/kg) at day 1, 5, 10, 15, 20 and 25. Finally, Cd concentration was measured by atomic absorption spectrophotometry in liver and kidney sections. Furthermore, pathological changes were investigated in these sections.

**Results:**

The results showed weight gain in Cd groups which received ascorbic acid and Se, in contrast to weight loss in parallel groups without vitamin C and Se. The stronger necrosis and inflammation have been observed in group received 5 mg/kg Cd compared to group with 1 mg/kg Cd (*P*<0.05). In addition, cadmium level was higher in untreated groups without any supplements, significantly (*P*<0.05).

**Conclusion:**

Drinking water with ascorbic acid may have prophylactic effects across cadmium, and combination of Se and ascorbic acid are associated with higher prophylactic effects in both kidney and liver in rats to decrease the Cd toxicity.

## Introduction

During recent years, industrial activities have been increased which leads to prolonged human exposure to industrial pollutant such as cadmium (Cd), which is used in batteries, pigments, plastic, electroplating, galvanism, refinery and petrochemical industries, and also arises from cigarette smoke (1-2). In addition, absorption of Cd in plants and animal's tissues is another way for human to get exposed with this pollutant. Because of genetic and epigenetic effects of Cd induced toxicity, bioindicators are under study for low levels of Cd as a water pollution (3).

Chronic exposure to Cd in human can cause damages to various organs such as lung, gastrointestinal, neurologic system, testis, immune system, endocrine and particularly kidneys and liver (4). Additionally, Cd can be carcinogen and also causes oxidation injuries in blood and other tissues which leads to cellular membrane dysfunction (5). Recent studies indicate that Cd can induce various genetic and epigenetic changes in plants and mammalian cells, both in vitro and in vivo (6). 

Chronic exposure to Cd often leads to kidneys dysfunction. The first nephrotoxic effect of Cd is increasing low molecular weight proteinuria (7). Seventy percent of filtrated Cd will be absorbed in proximal tubules and it will be concentrated in cortex which causes kidney dysfunction (8) and in addition, tubular injuries are less expected (9). Groten* et al.* has shown that urinary enzymes (Lactate dehydrogenase, Alkaline phopsphatase and N-acetyl-beta-D-glucosaminidase) will increase after 10 month exposure to Cd (10).

Liver is the next important target organ for Cd toxicity. After chronic Cd exposure of liver, increase of lipid peroxidation and changes of essential elements have been shown. In addition, Cd can disturb cellular respiration in liver (11). Thus, different pathologic changes in hepatocytes have been shown due to Cd toxicity which is distinguished with granulomatous inflammation, cellular reproduction, nodular hyper-plasia, apoptosis and necrosis. The morphologic changes arise after rising serum level of liver enzymes such as alanine aminotransferase (ALT), aspartate amino-transferase (AST) (12).

Different supplements have been used in various studies to adjust or compare with Cd toxicity. Ascorbic acid (Vitamin C) (13), vitamin E (14), aspirin (15) and zinc (5) have antioxidant characteristic and exert their preventive effect in a tissue with reduction of oxidative pressure. It is suggested that coenzyme Q10 and vitamin E play preventive role against the Cd exerted change in antioxidant defense system of the body (16). The next preventive substance is the beta-carotene which is used to adjust harmful effects of CdCl (2) in blood hematology and semen quality. This substance by itself or in combination with vitamin E could significantly reduce toxicity of Cd (17). Melatonin is another substance which is mentioned in several studies as modulation of Cd toxicity (4). Additionally, calcium suggested the modulatory effect of Cd induced toxicity (18). Furthermore, quercetin is proposed as preventive substance against kidney toxicity caused by Cd (9). It is also demonstrated that vitamin C reduces the accumulation of Cd in the tissue and thereby, it could exert advantageous effect due to the major accumulation of Cd in liver and kidney (19). Selenium (Se) is another essential element which its deficiency correlates with increase of lipid peroxidation in order to affect the function of the cell or its membrane (8). 

This study is aimed to investigate the effect of long term administration of combined Se and vitamin C treatment on prevention, reduction or adjustment of the chronic Cd induced toxicity in kidney and liver in rates.

## Materials and Methods

Animals

In this study, 60 adult male Wistar rats with primary weight of 140±15 g, were used. All the rats were maintained in an environment of 12 h light/12 h dark in the room temperature (22±3) with pellet rat diet. 

Experimental Design

One week prior to starting of the experiment, rats were weighed precisely (initial body weight). Then, the rats were randomly divided into 10 groups of six rats. 

The control group, got 1 mL sterile distilled water via gavage every other day and sham group, received 1 mL sterile distilled water via gavage every other day, accompanied by intraperitoneal (i.p.) injection of normal saline (1 cc/kg) for six times at the day 1, 5, 10, 15, 20 and 25 with access to Tao drinking water.

Supplemented drinking water was prepared with 10 mg/L of vitamin C, and Se (1 mg/kg) was administered via intraperitoneal injection for six times, during experiment, at the day 1, 5, 10, 15, 20 and 25. 

The rats of the eight experimental groups for 4 weeks, were exposed to: group 1 to 4 received 1 mg/kg cadmium chloride (CdCl(2)) in 1 mL sterile distilled water via gavage every other day, and group 1 exposed to Tao drinking water (1 mg/kg Cd). Group 2 exposed to supplemented drinking water (1 mg/kg Cd + supplemented water). Group 3 exposed to Se (1 mg Cd /kg + 1 mg Se /kg, i.p.). Group 4 exposed to both supplemented drinking water and Se (1 mg Cd/kg + 1 mg Se/kg, i.p. + supplemented water).

Group 5 to 8 received 5 mg/kg CdCl(2) in 1 mL sterile distilled water, via gavage every other day, and group 5 exposed to Tao drinking water (5 mg Cd/kg). Group 6 exposed to supplemented drinking water (5 mg Cd/kg + supplemented water). Group 7 exposed to Se (5 mg Cd/kg +1 mg Se/kg Se, i.p.), and group 8 exposed to supplemented drinking water and Se (5 mg Cd/kg + 1 mg Se/kg, i.p. + supplemented water).

Laboratory Assessment

After 24 hours from ending the interventions, rats were weighed. Then, rats were sacrificed in standard situation with inhalation of chloroform and their liver and left kidney were excised. 

Concentration of Cd in the liver and left kidney was determined by atomic absorption spectrophotometry (AAS) using a 400 SOUARES EUROMEX spectrophotometer (20). 

The liver and left kidney tissue were stained with hematoxylin and eosin, and analyzed using light microscope with 40X magnification view, for histological changes. The inflammation level of liver tissue was evaluated for portal inflammation, liver inflammation score, liver hydropic change score, liver necrosis, live granuloma, and the inflammation level of kidney tissue were assessed for presence of any inflammation or fibrosis in the kidney tissue and kidney fibrosis score was calculated also.

Statistical Analysis

Data are expressed as mean ± SD. The statistical analysis was based on five rats per group and mean values were compared by means of Student’s t-test at P-value<0.05. The Statistical Package of Social Science version 20.0 (SPSS, Chicago, Illinois, USA) was used for data analysis. 

## Results

Body weight changes and Cd concentration in liver and kidney tissues of 10 groups of study have been reported in Table 1. The highest amount of Cd is seen in liver and kidney tissues of group 5, receiving 5 mg Cd/kg alone (*P*<0.001 and *P*=0.04, respectively). 

**Table 1:** Mean body weight changes and mean cadmium concentration in the liver and kidney tissues

**Groups**	**Treatment**	**Initial body weight** **mean (g)**	**Final body weight** **mean(g)**	**Cd level in** **Liver (µg)**	**Cd level in** **Kidney (µg)**
**Control**		142	196	0	67
**Sham**		144	199	0	0
**1**	1 mg/kg Cd + tap water	140	173	1900	3167
**2**	1 mg/kg Cd + supplemented water*	133	189	1750	2583
**3**	1 mg Cd /kg + 1 mg Se /kg, i.p. + tap water	147	195	1500	2167
**4**	1 mg Cd/kg + 1 mg Se/kg, i.p. + supplemented water*	147	191	1373	1917
**5**	5 mg Cd/kg + tap water	135	152	2750	4167
**6**	5 mg Cd/kg + supplemented water*	142	194	2167	2750
**7**	5 mg Cd/kg +1 mg Se/kg Se, i.p. + tap water	132	183	2117	3083
**8**	5 mg Cd/kg + 1 mg Se/kg, i.p. + supplemented water*	149	196	1833	2083

*supplemented with 10 mg/l vitamin C.

i.p.: intraperitoneal; Cd: cadmium; Se: Selenium.

**Table 2:** Different grades of focal (spotty) lytic necrosis, apoptosis, and focal inflammation in the liver tissue

Grade		Groups									
		Control	Sham	1	2	3	4	5	6	7	8
Absent	No.	5	4	0	0	1	0	0	0	0	2
	%	30.2	57.1	-	-	14.3	-	-	-	-	28.6
Mild	No.	1	2	1	2	3	2	0	0	2	4
	%	12.5	12.5	6.2	12.5	18.8	12.5	-	-	12.5	25.0
Moderate	No.	0	0	1	4	1	3	2	5	4	0
	%	-	-	5.0	20.0	5.0	15.0	10.0	25.0	20.0	-
Marked	No.	0	0	4	0	1	1	4	1	0	0
	**%**	-	-	36.4	-	9.1	9.1	36.4	9.1	-	-

Absent: none, Mild (One focus or less per 10 objectives), Moderate (Two to four foci per 10Xobjectives or five to ten foci per 10Xobjectives), Marked (More than ten foci per 10Xobjectives)

Overall, consumption of supplemented water was 11 mg/animal vitamin C in group 2 (1 mg/kg Cd + Tao water), 10.1 mg/animal vitamin C in group 4 (1 mg Cd/kg + 1 mg Se/kg, i.p. + supplemented water), 9.2 mg/animal vitamin C in group 6 (5 mg Cd/kg + supplemented water), and 10.2 mg/animal vitamin C in group 8 (5 mg Cd/kg + 1 mg Se/kg, i.p. + supplemented water). 

Table 2 shows different grades of focal or spotty necrosis in liver tissue in experimental groups. As seen the highest grades were obtained in groups receiving Cd alone (*P*<0.001). Vitamin C and Se could alleviate the necrosis in experimental groups.Table 3 shows different grades of portal infla-mmation in experimental groups. As seen the highest grades were found in groups receiving Cd alone (*P*=0.02). 

Table 4 shows different grades of liver congestion in experimental groups. As noted, the highest grades were found in groups receiving Cd alone (*P*<0.001).

Table 5 shows different grades of liver parenchymal inflammation in experimental groups. As seen the highest grades of inflammation were presented in groups receiving Cd alone but this difference was not significant (*P*=0.65).

Different grades of inflammation in kidney tissue of experimental groups, is shown in Table 6. As seen the highest grade of inflammation was observed in groups receiving Cadmium alone (*P*=0.01). 

**Table 3:** Different grades of portal inflammation in the liver tissue

Portal Inflammation Grade		Groups									
		Control	Sham	1	2	3	4	5	6	7	8
None	No.	4	3	0	0	1	0	0	0	0	1
	%	20.0	60.0	-	-	20.0	-	-	-	-	20.0
Rare	No.	2	3	1	2	4	3	0	1	2	4
	%	10.0	15.0	5.0	10.0	20.0	15.0	-	5.0	10.0	20.0
Mild	No.	0	0	1	3	1	3	0	3	4	1
	%	-	-	6.2	18.8	6.2	18.8	-	18.8	25.0	6.2
Moderate	No.	0	0	3	1	0	0	4	2	0	0
	%	-	-	30.0	10.0	-	-	40.0	20.0	-	-
Marked	No.	0	0	1	0	0	0	2	0	0	0
	%	-	-	33.3	-	-	-	66.7	-	-	-

None: No portal inflammation, Rare (scant/ mild inflammation in some portal areas), Mild (mild to moderate inflammation in some/most portal areas), Moderate (moderate inflammation in all portal areas, marked (marked inflammation in all portal areas)

**Table 4:** Different grades of liver congestion

Liver Congestion Grade		Groups									
		Control	Sham	1	2	3	4	5	6	7	8
None	No.	5	2	0	0	0	0	0	0	0	3
	%	-	-	-	-	-	-	-	-	-	-
Rare	No.	1	4	0	0	2	2	0	0	2	3
	%	15.4	30.8	-	-	15.4	15.4	-	-	15.4	23.1
Mild	No.	0	0	1	4	3	2	0	4	3	0
	%	-	-	5.9	23.5	17.6	11.8	-	23.5	17.6	-
Modreate	No.	0	0	3	2	1	2	3	2	1	0
	%	-	-	21.4	14.3	7.1	14.3	21.4	14.3	7.1	-
Marked	No.	0	0	2	0	0	0	3	0	0	0
	%	-	-	40.0	-	-	-	60.0	-	-	-

None: no sinusoidal congestion, Rare (Rare foci), Mild (congestion in one third of liver parenchyma), Moderate (between one third to two third of liver parenchyma), Marked (more than two third of liver parenchyma)

**Table 5:** Different grades of liver parenchymal inflammation (confluent necrosis) *.

Liver Parenchyma Inflammation Grade		Groups									
		Control	Sham	1	2	3	4	5	6	7	8
Absent	No.	6	5	1	2	2	3	0	5	5	5
	%	7.1	17.9	3.6	7.1	7.1	10.7	-	17.9	17.9	17.9
Rare	No.	0	1	3	2	3	3	3	1	1	1
	%	-	5.6	16.7	11.1	16.7	16.7	16.7	5.6	5.6	5.6
Mild	No.	0	0	2	2	1	0	3	0	0	0
	%	-	-	25.0	25.0	12.5	-	37.5	-	-	-
Moderate to severe	0	0	0	0	0	0	0	0	0	0	0

Absent: 0, Rare (Focal confluent necrosis), Mild (Zone 3 necrosis in some areas), Moderate to severe (the spectrum from more diffuse and severe necrosis/ inflammation to panacinar necrosis)

* we did not have the following items (more severe inflammation), you can add a row with 0 number or percent: Zone 3 necrosis in most areas, Zone 3 necrosis 1 occasional portal-central (P-C) bridging, Zone 3 necrosis 1 multiple P-C bridging, and Panacinar or multiacinar necrosis.

**Table 6:** Different grades of inflammation in the kidney tissue

Grade		Groups									
		Control	Sham	1	2	3	4	5	6	7	8
Rare	No.	4	5	0	1	2	0	0	1	2	4
	%	6.7	33.3	-	6.7	13.3	-	-	6.7	13.3	26.7
Mild	No.	2	1	2	3	1	4	1	3	3	2
	%	5	5.0	10.0	15.0	5.0	20.0	5.0	15.0	15.0	10.0
Moderate	No.	0	0	3	2	3	1	2	2	1	0
	%	-	-	21.4	14.3	21.4	7.1	14.3	14.3	7.1	-
Marked	No.	0	0	1	0	0	1	3	0	0	0
	%	-	-	20.0	-	-	20.0	60.0	-	-	-

Rare: in rare foci, Mild (inflammatory cell infiltration was identified in one third of kidney parenchyma), Moderate (In one third to two third of kidney parenchyma), Marked (More than two third of the kidney parenchyma was involved by inflammatory cell infiltration)

## Discussion

Chronic exposure to Cd, may lead to nephrotoxicity, hepatotoxicity, damages to nervous system, immune and endocrine system, and finally cancer in human beings (21-22) Moreover, accumulation of heavy metals may affect the function of cells’ proteins which can lead to organ dysfunction (15). Present study confirmed Cd-induced inflammation, in kidney and liver of rats, after 28 days exposure to 1 or 5 mg Cd/ kg, which adjusted by combination of Vitamin C and Se. This adjustment prevents Cd induced weight loss, significantly. 

Omonkhua* et al.* Study showed that low dosages of Cd (1 to 3 μg/kg BW), for four weeks, induce Cd toxicity in rats, with or without vitamin C supplementation. Serum alkaline phosphatase had increased significantly in all the groups, even in groups supplemented with vitamin C. But, for some other evaluated parameters, such as serum calcium and bone protein concentration, vitamin C prevented the effects of Cd on the treated rats, indicating that vitamin C may have protective effect, even against low dosage of Cd (13).

In a study, vitamin C supplement was used for adjustment Cd toxicity. In this study, rats were fed with supplemented food with 10 mg/kg of Cd, equivalent with 1.0-1.2 mg Cd/kg in 28 days. Vitamin C was added to one of the experimental group's drinking water. The result of this study showed less concentration of Cd in liver, kidneys, testis and muscles in this group. Thus, this group had better weight gain at the end of the experimental period (23) but mentioned study did not evaluate the prophylactic effects of vitamin C on high dosages of Cd, which was evaluated in present study. 

El-Sharaky* et al.* studied on the adjustment of Cd toxicity in rat by using Se. In this study intraperitoneal injection of 2 mg/kg Cd for 10 days leads to lipid peroxidation in rat's kidneys. Using 1 mg/kg Se twice a day protected the kidney tissue via increasing the antioxidant effect (8). By using 1mg/kg Se, increase of antioxidant effect leads to decrease hepatotoxicity of Cd (21).

 In a study of protective effect of vitamin E and aspirin towards the cell toxicity, Mattie* et al.* confirmed that these agents exert their protective effect via antioxidation (15). Administration of intraperitoneal injection of CdCl(2) with 0.4 mg/kg concentration caused the changes in antioxidation defense system. To adjust this effect, intramuscular injection of 10 mg/kg coenzyme Q (10) with 20 IU/kg vitamin E were used. Interestingly, these results indicated that coenzyme Q (10) and vitamin E could reduce the toxicity of high dosage of Cd (16). Furthermore, many other substances such as, vitamin E 100 mg/kg and beta-carotene 10 mg/kg independently or in combination have been used in rats via gavages to adjust the toxic effect of 5 mg/kg of CdCl(2); as a result, increase in level of liver enzymes, creatinine, urea, bilirubin and decrease in hemoglobin and albumin have been seen which this adverse effect reduced by combination of alpha-tocopherol and beta-carotene (19). 

Beneficial effects of vitamin C were evaluated in several studies, to reduce the Cd induced toxicity. Present study revealed that vitamin C supplement caused decrease in the Cd content of liver and kidney, especially vitamin C in combination with Se, reduced Cd content, more than distinct administration of Se or vitamin C. In a study on Cd-induced toxicity, administration of vitamin C was led to decrease in lipid peroxidation and this study showed that vitamin C could play protective role against the testicular steroidogenesis and germ cell death (24). In Grosicki study, 1-1.2 mg/kg CdCl(2) daily dosage were administrated for 28 days, in order to pose the rats in combination with 1.5 mg/L vitamin C via gavage. Eventually, accumulation of Cd in liver, kidney, testes, and muscles has been reduced by using vitamin C and the treated group had appropriate weight gain during the experiment (23). Additionally, in another study of vitamin C was used to prevent the lethality effect of acute Cd toxicity; administration of 25 μmol/kg s.c of CdCl(2) after 72 hours cause 93% mortality in rats which in comparison with other treated groups with vitamin C (2 g/kg s.c.) the killing effect was almost inhibited (21). 

In present study, a significant decrease was observed in Cd level of Se treated groups, especially when Se was administered in combination with vitamin C. El-Sharaky* et al.* showed that intraperitoneal administration of 2 mg/kg CdCl(2) in 10 days led to lipid peroxidation in kidneys of the animals; remarkably, administration of 1mg/kg of Se, twice a day, resulted in protection of kidney and decrease in hepatotoxicity by increase in activity of antioxidant enzymes (8). In another study administration of 1 mg of CdCl(2) in a guinea pig per day via drinking water has been evaluated for lipid peroxidation. After following the experiment, increase in lipid peroxidation in kidney and liver has been demonstrated. However, this effect was reduced after administration of daily 100 mg of vitamin C. It is suggested that usefulness of vitamin C, is dependent on amount of accumulated Cd (25), but beneficial effects of Se have not been reported, as a dependent effect with Cd accumulated levels. In addition, recent study reported Se as a chemopreventive and chemo-therapeutic agents for human cancers and daily supplement of Se has been recommended (26). Therefore, in this study, new combination of potent prophylactic agents were studied (vitamin C and Se), which do not have major side effects in prolonged daily usage. 

## Conclusion

Based on the findings of this study, CdCl accumulates and influences on liver and kidney tissues pathologically and by using supplements such as vitamin C and Se, these effects can be subsided. In future clinical trials on humans, toxicity of this heavy metal can be evaluated with the addition of supplements containing Se and vitamin C in daily drink and food of workers of many industries such as battery and paint manufacturing, and in case of similar results in human, by this way, by decrease of Cd toxicity complications, national health costs will be reduced; Vitamin C and Se are existed in a common daily drink, such as non-alcoholic beer.
